# Wide dissemination of Gram-negative bacteria producing the taniborbactam-resistant NDM-9 variant: a One Health concern

**DOI:** 10.1093/jac/dkad210

**Published:** 2023-07-03

**Authors:** Christophe Le Terrier, Patrice Nordmann, Chloé Buchs, Doris Yoong Wen Di, Gian Maria Rossolini, Roger Stephan, Mariana Castanheira, Laurent Poirel

**Affiliations:** Emerging Antibiotic Resistance, Medical and Molecular Microbiology, Department of Medicine, University of Fribourg, Chemin du Musée 18, CH-1700 Fribourg, Switzerland; Division of Intensive Care Unit, University Hospitals of Geneva, Geneva, Switzerland; Emerging Antibiotic Resistance, Medical and Molecular Microbiology, Department of Medicine, University of Fribourg, Chemin du Musée 18, CH-1700 Fribourg, Switzerland; Swiss National Reference Center for Emerging Antibiotic Resistance, Fribourg, Switzerland; Institute for Microbiology, Lausanne University Hospital and University of Lausanne, Lausanne, Switzerland; Emerging Antibiotic Resistance, Medical and Molecular Microbiology, Department of Medicine, University of Fribourg, Chemin du Musée 18, CH-1700 Fribourg, Switzerland; School of Earth Sciences and Environmental Engineering, Gwangju Institute of Science and Technology, Gwangju 61005, Republic of Korea; Clinical Microbiology and Virology Unit, Florence Careggi University Hospital, Florence, Italy; Department of Experimental and Clinical Medicine, University of Florence, Florence, Italy; Institute for Food Safety and Hygiene, Vetsuisse Faculty, University of Zurich, Winterthurerstrasse 272, CH-8057 Zurich, Switzerland; JMI Laboratories, North Liberty, IA, USA; Emerging Antibiotic Resistance, Medical and Molecular Microbiology, Department of Medicine, University of Fribourg, Chemin du Musée 18, CH-1700 Fribourg, Switzerland; Swiss National Reference Center for Emerging Antibiotic Resistance, Fribourg, Switzerland

The increasing trend of carbapenem resistance observed in Gram-negative bacteria is mainly related to the dissemination of carbapenemase-encoding genes.^1^ A particular threat are those encoding MBLs, since production of MBLs leads to very limited treatment options.^1^ MBLs of the NDM group have been largely disseminated in human, animals and in the environment, making them the most frequently identified acquired carbapenemases worldwide.^1,2^ NDM-like β-lactamases hydrolyse all β-lactams (BLs) except monobactams, and are not inactivated by most of the recently developed β-lactamase inhibitors (BLIs) (avibactam, relebactam, vaborbactam, nacubactam or zidebactam).^3^ The newly developed cyclic boronate BLI, taniborbactam, alias VNRX-5313, is one of the few BLIs possessing significant inhibitory activity against MBLs, with the exception of IMP-like enzymes, and is currently in clinical development in combination with cefepime (https://clinicaltrials.gov/ct2/show/NCT03840148).^4^ Hence, the combination cefepime/taniborbactam displays excellent *in vitro* activity against NDM-producing Gram-negative isolates worldwide.

However, using a panel of isogenic recombinant *Escherichia coli* strains producing a variety of MBLs, including NDM enzymes, we recently showed that NDM-9, differing from NDM-1 by a single amino acid substitution (E152K), was not inhibited by taniborbactam.^5^

Here we report the *in vitro* activity of cefepime/taniborbactam in comparison with other recently developed BL/BLI combinations against a collection of NDM-9 producers. Our collection included four different bacterial species: *E. coli*, *Klebsiella pneumoniae*, *Klebsiella variicola* and *Acinetobacter baumannii*, recovered either from human or water origins and from four different countries (France, Switzerland, South Korea, USA) located in three different continents.

Susceptibility testing was performed by broth microdilution and interpreted according to the EUCAST guidelines for cefepime, aztreonam, ceftazidime, ceftazidime/avibactam, imipenem, imipenem/relebactam, meropenem, meropenem/vaborbactam and cefiderocol (using an iron-depleted medium for the latter). Susceptibility testing with BL/BLI combinations including cefepime/taniborbactam, cefepime/zidebactam and meropenem/nacubactam were interpreted according to the breakpoint criteria for the BL alone. Zidebactam, avibactam, nacubactam, relebactam and taniborbactam were tested at a fixed concentration of 4 mg/L, whereas vaborbactam was tested at 8 mg/L.^6^ Susceptibility testing of nacubactam and zidebactam were also determined alone, as well as at a 1:1 ratio with their respective BL partner (cefepime/zidebactam 1:1 and meropenem/nacubactam 1:1) considering the significant antibacterial activity of those BLIs.^6^*E. coli* ATCC 25922 was used as a WT reference strain.

As expected for NDM-producing isolates, they all showed high resistance to ceftazidime, ceftazidime/avibactam, cefepime, imipenem and imipenem/relebactam, and a reduced susceptibility to meropenem and meropenem/vaborbactam (Table 1). However, all NDM-9 producers displayed high MICs of cefepime/taniborbactam.^5^ Interestingly, MICs of combinations including zidebactam either at a fixed concentration or at a 1:1 ratio with cefepime were very low for *E. coli* strains (≤0.5 mg/L), although high (8 mg/L) in *Klebsiella* spp. and *A. baumannii* (>32 mg/L). These results are in line with the MICs observed for zidebactam alone in these different species, therefore highlighting the potent antibacterial activity of that BLI (Table 1).^6^ Similarly, combinations including nacubactam, namely meropenem/nacubactam (4 mg/L) or meropenem/nacubactam 1:1, showed higher MICs when testing *A. baumannii* and *Klebsiella* spp. than for *E. coli*, also highlighting the direct antibacterial activity of nacubactam, as previously published.^6^

**Table 1.** Susceptibility testing of NDM-9-producing isolates for the different BL/BLI combinations tested

Strain					MICs (mg/L)^a^																
	ST	Country of isolation/ and year	Origin	BL(s)	CAZ	CZA	FEP	FEP-TAN	FEP-ZID	FEP-ZID 1:1	IMP	I/R	MEM	MVB	MEM-NAC	MEM-NAC 1 :1	ATM	AZA	ZID	NAC	FDC
*E. coli*	167	USA 2015	Clinical	NDM-9, CTX-M-65	>256	>128	>256	>128	≤0.125	0.25	>256	>128	64	64	≤0.125	1	>128	4	0.5	1	>64
*E. coli*	167	USA 2015	Clinical	NDM-9, CTX-M-65, TEM-1	>256	>128	>256	>128	≤0.125	0.125	>256	>128	32	32	≤0.125	1	>128	≤0.125	0.25	1	32
*K. pneumoniae*	147	Switzerland 2018	Water	NDM-9, SHV-11, CTX-M-15, OXA-9, TEM-1	>256	>128	256	128	4	4	128	128	16	16	16	4	>128	≤0.125	8	8	0.125
*K. pneumoniae*	147	Italy 2020	Clinical	NDM-9, CTX-M-15, OXA-1, OXA-9, TEM-1A	>256	>128	>256	128	0.5	0.5	128	128	8	8	8	8	>128	0.5	0.5	8	2
*K. variicola* GJ1	363	South Korea 2016	Water	NDM-9, LEN-13	>256	>128	128	128	8	8	256	>128	32	32	16	16	>128	≤0.125	>8	>8	0.25
*K. variicola* GJ2	363	South Korea 2016	Water	NDM-9, LEN-13, TEM-1B	>256	>128	128	128	4	4	>256	>128	32	32	32	16	128	≤0.125	>8	>8	0.25
*K. variicola* GJ3	363	South Korea 2016	Water	NDM-9, LEN-13, CTX-M-65, TEM-1B	>256	>128	128	128	4	4	>256	>128	32	32	16	16	128	≤0.125	>8	>8	0.25
*A. baumannii*	52	Switzerland 2021	Clinical	NDM-9, OXA-58	>256	>128	>256	>128	>128	>32	>256	>128	128	128	128	>32	128	128	>8	>8	1
*K. pneumoniae*	147	Switzerland 2022	Clinical	NDM-1, TEM-1, OXA-9, CTX-M-224, CTX-M-54	>256	>128	>256	1	0.25	0.25	8	8	8	8	≤0.125	2	≤0.25	≤0.125	0.5	2	1
*E. coli* ATCC 27922	NA	—	—	—	≤0.25	≤0.125	≤0.25	≤0.125	≤0.125	≤0.03	≤0.25	0.25	≤0.25	≤0.125	≤0.125	≤0.03	≤0.25	≤0.125	0.06	1	≤0.06

, no BL; ZID, zidebactam; NAC, nacubactam, FEP-ZID 1:1, cefepime/zidebactam at 1:1 ratio; MEM-NAC 1:1, meropenem/nacubactam at 1:1 ratio.

CAZ, ceftazidime; CZA, ceftazidime/avibactam; FEP, cefepime; FEP-ZID, cefepime/zidebactam; FEP-TAN, cefepime/taniborbactam; IMP, imipenem; I/R, imipenem/relebactam; MEM, meropenem; MVB, meropenem/vaborbactam; MEM-NAC, meropenem/nacubactam; ATM, aztreonam; AZA, aztreonam/avibactam; FDC, cefiderocol. In those BL/BLI combinations, zidebactam, nacubactam, relebactam, avibactam were used at fixed concentration of 4 µg/mL. Vaborbactam were used at fixed concentration of 8 µg/mL.

Among these NDM-9 producers, aztreonam/avibactam remained highly effective against all Enterobacterales strains, except for a single *E. coli* isolate, likely related to a PBP3 modification, as previously reported.^7^ Cefiderocol also showed high activity against those NDM producers, expect for the two *E. coli* strains. Of note, the NDM-9-producing *A. baumannii* was resistant to all BLs and recently developed BL/BLI combinations, and with an MIC of cefiderocol at 1 mg/L (currently no available EUCAST breakpoint for that species).

This study firstly highlights the *in vitro* ineffectiveness of cefepime/taniborbactam against NDM-9-producing isolates, regardless of the species of concern. It highlights that NDM-9-producing Gram-negative isolates are already circulating worldwide, even though such a last-line BL/BLI combination is still not commercially available. In addition, some other worldwide reports indicated a large variety of bacterial species that includes *E. coli*, *Klebsiella aerogenes*, *K. pneumoniae*, *K. variicola*, *Cronobacter sakazakii* and *A. baumannii* as carriers of the *bla*_NDM-9_ gene. They have been recovered from humans but also from animals (chickens) and the environment (rivers), and in many different countries including China, French Polynesia, Italy, South Korea, Tunisia and Switzerland.^8–10^ Of particular concern is the report of an MDR NDM-9-producing ST147 *K. pneumoniae* (included in this study) that was clonally related to other NDM-1-producing *K. pneumoniae* isolates being part of a nosocomial outbreak involving patients hospitalized in the same region of Italy.^10^ It remains to determine what kind of selection factor(s) might have driven such NDM-1 to NDM-9 evolution.

We show here that the future effectiveness of cefepime/taniborbactam, but also of any other BL/BLI combination supposed to include taniborbactam as BLI, might be compromised by the circulation of the NDM-9 enzyme. Worryingly, the potential of the NDM-9-encoding gene to successfully spread among many different species and many different environments is proven here, as a good example of a One Health critical issue.

## Data Availability

All data from this study can be made available upon request, without limitation in time.
